# Examining the Discontinuation of Clozapine Due to Serious Side Effects Over A Decade at a University Hospital Inpatient Clinic

**DOI:** 10.1192/j.eurpsy.2025.412

**Published:** 2025-08-26

**Authors:** D. Koçyiğit, R. Aktaş, S. Çoşkun, E. Özçelik Eroğlu, E. Mutlu, A. E. Anıl Yağcıoğlu

**Affiliations:** 1 Department of Psychiatry, Hacettepe University, Ankara, Türkiye

## Abstract

**Introduction:**

Clozapine stands out among all other antipsychotics due to its superior efficacy in treatment resistant schizophrenia. Despite its proven superiority, it is not the first antipsyhotic choice on grounds of serious side effects. Clozapine remains underutilized, primarily due to its troubled safety profile.Lower prescription rates may be related with physicians’ hesitation because of side effects rather than patients’ unwillingness to use the drug.

**Objectives:**

To elaborate on the life-threatening side effects and discontinuation of clozapine, we retrospectively reviewed the medical records of all patients admitted to the inpatient psychiatry unit at the Hospital of Hacettepe University Faculty of Medicine, Ankara, Türkiye between January 2010 and April 2022.

**Methods:**

Hospital records of inpatients with psychotic disorders, identified by ICD-10 codes (F20.X, F25.X, F.22, F28, F29), and patients with bipolar affective disorder, identified by ICD-10 code (F31.X) who discontinued clozapine during their hospitalization due to serious side effects were thoroughly investigated.

**Results:**

Among a total of 2298 patients hospitalized during the specified period, 178 patients with psychotic disorders and 21 patients with bipolar affective disorder were clozapine users. In this sample, 14 patients with psychotic and 3 patients with bipolar affective disorders had a serious side effect due to clozapine which led to discontinuation in 15 patients (7.53%). The median age of the cases was 32-years (min-max:18-62), the median duration of illness was 10-years (min-max:2-30), and the mean clozapine dose at onset of the serious side effect was 245±149.95 mg/day. The observed serious side effecs associated with clozapine included myocarditis (n=10, 58.8%), agranulocytosis (n=3, 17.6%), neutropenia (n= 1, %5.88), pancreatitis and myocarditis (n=1, 5.88%), refractory increase in level of C-reactive protein (n=1, 5.88%), refractory constipation, weight gain and worsening in obsessive-compulsive symptoms (n=1, 5.88%), and suspicion of neurotoxicity of clozapine in a patient with mutation in multi-drug resistance-1 gene (n=1, 5.88%). There was no report of sudden death, cardiac arrest or need for intensive care unit. The majority of serious side effects (88.2%) occurred within the first 6 months of clozapine initiation. Two patients (11.7%) were able to continue clozapine with clinical management. Among patients who discontinued clozapine, 3 (20%) were rechallenged with clozapine in which 2 attempts (66,6%) were successful.

**Conclusions:**

An examination of 12 years of inpatient clozapine treatment at a University Hospital clinic revealed that even life-threatening side effects of clozapine can be managed successfully with close clinical care. Physicians’ concern about serious side effects should not lead to underutilization of clozapine in patients who could benefit from its trial.

**Disclosure of Interest:**

D. Koçyiğit: None Declared, R. Aktaş: None Declared, S. Çoşkun: None Declared, E. Özçelik Eroğlu: None Declared, E. Mutlu: None Declared, A. E. Anıl Yağcıoğlu Grant / Research support from: Janssen, Boehringer Ingelheim, Consultant of: Janssen, Boehringer Ingelheim, Abdi Ibrahim Otsuka, Paid Instructor of: Abdi Ibrahim Otsuka, Speakers bureau of: Abdi İbrahim, Nobel, Janssen, Santa Farma.

